# Intratumoral gene delivery of 4-1BBL boosts IL-12-triggered anti-glioblastoma immunity

**DOI:** 10.1101/2025.02.03.636330

**Published:** 2025-02-05

**Authors:** Taral R. Lunavat, Lisa Nieland, Sanne M. van de Looij, Alexandra J.E.M. de Reus, Charles P. Couturier, Chadi A. El Farran, Tyler E. Miller, Julia K. Lill, Maryam Schübel, Tianhe Xiao, Emilio Di Ianni, Elliot C. Woods, Yi Sun, David Rufino-Ramos, Thomas S. van Solinge, Shadi Mahjoum, Emily Grandell, Mao Li, Vamsi Mangena, Gavin P. Dunn, Russell W. Jenkins, Thorsten R. Mempel, Xandra O. Breakefield, Koen Breyne

## Abstract

The standard of care in high-grade gliomas has remained unchanged in the past 20 years. Efforts to replicate effective immunotherapies in non-cranial tumors have led to only modest therapeutical improvements in glioblastoma (GB).

Here, we demonstrate that intratumoral administration of recombinant interleukin-12 (rIL-12) promotes local cytotoxic CD8 ^POS^ T cell accumulation and conversion into an effector-like state, resulting in a dose-dependent survival benefit in preclinical GB mouse models. This tumor-reactive CD8 T cell response is further supported by intratumoral rIL-12-sensing dendritic cells (DCs) and is accompanied by the co-stimulatory receptor 4-1BB expression on both cell types. Given that DCs and CD8 ^POS^ T cells are functionally suppressed in the tumor microenvironments of *de novo* and recurrent glioma patients, we tested whether anti-tumor response at the rIL-12-inflamed tumor site could be enhanced with 4-1BBL, the ligand of 4-1BB. 4-1BBL was delivered using an adeno-associated virus (AAV) vector targeting GFAP-expressing cells and resulted in prolonged survival of rIL-12 treated GB-bearing mice.

This study establishes that tumor antigen-specific CD8 T cell activity can be directed using an AAV-vector-mediated gene therapy approach, effectively enhancing anti-GB immunity.

## Full Text Availability

The license terms selected by the author(s) for this preprint version do not permit archiving in PMC. The full text is available from the preprint server.

